# COVID-19 Humic/Fulvic Acid Plus Epigallocatechin Gallate Treatment: A Retrospective Chart Review

**DOI:** 10.7759/cureus.77188

**Published:** 2025-01-09

**Authors:** Richard Williams

**Affiliations:** 1 Geriatrics, Edward Via College of Osteopathic Medicine, Blacksburg, USA

**Keywords:** covid-19, egcg, humic/fulvic avid, integrative medicine, nursing home, vitamin c

## Abstract

Introduction:The initial COVID-19 infection caused significant mortality in the nursing home setting. The mortality in our facility was approximately 50% and occurred in the later phase of the initial infection. In the nursing home setting, there were no approved treatment regimes. Specific treatment was left to the treating physician in concert with the administrative medical officials; immunizations were approved in December 2020. Concurrent publications indicated that epigallocatechin gallate (EGCG) and humic/fulvic acid with vitamin C possess the ability to interfere with the SARS-CoV-2 virus; both can inhibit viral attachment and reproduction, along with providing significant anti-inflammatory activity. They also exhibit good safety profiles and were previously utilized in our facility. With the high mortality and significant published data on inhibitory aspects of EGCG, humic/fulvic acid, and vitamin C, our facility utilized these supplements to treat COVID-19-positive patients.

Methods: This retrospective chart review analyzes the effectiveness of an integrative treatment consisting of EGCG and humic/fulvic acid with vitamin C provided to COVID-19 patients in a long-term care facility between October and December 2020. Nursing home patients with COVID-19 infections were either provided integrative treatment utilizing EGCG, humic/fulvic acid, and vitamin C or provided care without integrative support. All EGCG patients were informed, and, when needed, the first line of authority, power of attorney (POA) was contacted and informed that the integrative approach was not standard treatment (but was reviewed and approved by Pulaski Health and Rehabilitation Medical Facility directors and the patient's physician). Patients were informed of the safety literature and compounds' utilization in viral infections prior to accepting treatment.

Results: A review of 60 records indicates that among 22 patients receiving treatment with the integrative combination, there were no mortalities. Among 38 patients treated without this integrative support, there were 21 deaths.

Conclusions: In a retrospective review of the treatment data, treatment of COVID-19 in the nursing home setting with EGCG, humic/fulvic acid, and vitamin C resulted in a significant reduction in overall mortality.

## Introduction

According to data from the COVID Tracking Project (CTP), from March 7, 2020, to March 7, 2021, deaths among residents of long-term care facilities, including nursing homes, assisted living, and other long-term care facilities, made up over a third of all US COVID-19 deaths [1]. There were no approved treatments for patients in long-term care facilities during the data collection period of the CTP; see the methods section below. Epigallocatechin gallate (EGCG) and humic/fulvic acid with vitamin C have been demonstrated to interfere with various viral infections, with recent docking studies showing activity against COVID-19 in attachment and reproduction [2]. The polyphenols also had better docking scores than remdesivir (Gilead Sciences) and favipiravir (Fujifilm Toyama Chemical) when docked with RNA-dependent RNA polymerase (RdRp). These results suggest the potential benefit of tea polyphenols in treating COVID-19. Humic/fulvic has also been shown to inhibit the COVID-19 virus in both attachment and replication [3,4]. Both treatments provide anti-inflammatory activity that might be useful in treating COVID-19 infection sequelae. Given these compounds' longstanding safety profiles, ease of utilization, and previous use in the facility, they were utilized in our facility to treat COVID-19 patients.

## Materials and methods

Materials

All materials were approved by the United States Pharmacopeia (USP) and/or Current Good Manufacturing Practice (CGMP). All materials were given at 7:00 a.m., one hour before breakfast, and at 7:00 p.m., one hour after supper.

Humic Bio-Mass Vegetal Minerals 262.9 mg/30 ml (Vital Earth Minerals)

Dosage: 30 cc; if fever, myalgias, and wheezing, 60 cc. "Humic/fulvic acids are natural and water-soluble polymers and key ingredients of humic substances. They are defined as a series of high molecular weight substances, yellow to black in color, formed by secondary synthesis reactions. They are complex substances present in soil and plants in trace amounts" [4]. "Humate is well tolerated with an excellent safety profile and merits further evaluation in patients suffering from inflammatory conditions" [5]. 

Decaffeinated Green Tea Extract (GTE) (Leaf) 725mg, Standardized to 98% Polyphenols (710.5 mg), 75% Catechins (543.75 mg), 45% Epigallocatechin-3-gallate (EGCG)(326.5 mg) (Vitacost)

Dosage: One tablet. "The compound (-)-epigallocatechin-3-gallate (EGCG) is the major catechin found in green tea *Camellia sinensis*. This polyphenolic compound and several related catechins are believed to be responsible for the health benefits associated with the consumption of green tea"[6].

Vitamin C (Ascorbic Acid) 250 mg (Vitacost)

Vitamin C, also known as L-ascorbic acid, is a water-soluble vitamin that is naturally present in some foods, added to others, and available as a dietary supplement. It is also an important physiological antioxidant and plays a vital role in immune function [7]. Vitamin C, when ingested with EGCG, increases the concentration and stability of EGCG [8]. 

Methods

The study sample included 60 charts for patients receiving care at the Pulaski Health and Rehabilitation Center between October 17, 2020, and December 30, 2020. Inclusion criteria for the retrospective chart review included patients diagnosed with and treated for COVID-19. Charts meeting the inclusion criteria were divided into an integrative treatment group consisting of patients who were provided the integrated treatment described above and a control/nonintegrative group consisting of patients who were provided treatment using a nonintegrative approach. Exclusion criteria included no COVID-19 diagnosis. Patient charts meeting inclusion criteria were assigned a number; the only recorded information was patient age, calculated mortality and risk of the COVID-19 infection, and physician notes indicating symptomatic status or coinfection; no other identifying data were recorded. The data were retrospectively reviewed and calculations for mortality and risk were performed.

COVID-19 Diagnosis

All residents were screened twice weekly as part of a routine screening profile and tested if COVID-19 symptoms developed. Rapid tests (BinaxNOW COVID-19 Antigen, Abott) were obtained, and if positive, a repeat test was sent to the state lab for polymerase chain reaction (PCR) analysis. Patients with a positive COVID test were transferred to the isolation unit and treated with either integrative or nonintegrative therapy.

Facility COVID-19 Treatment Details

Medical physicians practicing at this facility included a board-certified physician in internal and integrative medicine and another physician certified in internal medicine. Upon admission to the facility, patients were randomly assigned to either physician unless specific physicians were requested. The integrative treatment approach was initiated by the integrative physician in the facility. COVID-19 patients under the care of the integrative physician were treated with an integrative protocol consisting of GTE with EGCG, humic/fulvic acid, and vitamin C. The integrative treatment approach was reviewed and approved for use and publication by the Vice President of Clinical Services and the Corporate Medical Administrator of Pulaski Health and Rehabilitation Center. All patients offered the integrative treatment approach were informed that the regime was not standard therapy but had been reviewed by their physician and corporate officials and approved for utilization at the Pulaski Health and Rehabilitation Center. If the patient was unable to give appropriate consent for treatment, the patient's power of attorney (POA) was contacted, and the specifics of the treatment approach were reviewed and approved by the patient's POA. COVID-19 patients under the care of the other physician received a nonintegrative approach. There was no approved standard of care for the treatment of COVID-19 in the nursing home at this time. The use of corticosteroids and antibiotics, if pneumonia was diagnosed, was suggested [9]. No corticosteroids were utilized in the integrative treatment approach, and three patients required antibiotics for fever and pneumonia. In the nonintegrative group, patients were given methylprednisolone 32 mg and antibiotics (personal conversation).

Statistical analysis

All demographics and research variables were summarized by treatment group using descriptive measures of central tendency and dispersion. For continuous variables, a measure of central location using the sample mean and dispersion by standard deviation was utilized. Categorical variables utilized proportions. Fisher's exact test examined the association between death and treatment arm categorical variables. For continuous outcomes, two sample tests assuming unequal variance were used to compare treatment groups on age, mortality risk, and risk scores. Careful attention was paid to the normality assumption, which was examined using histograms and normal probability plots. Because the normality assumption appears violated, the nonparametric Wilcoxon Rank Sum procedures were employed. To test the association between death and treatment group while adjusting for age, exact logistic regression models were constructed. All tests were conducted using SAS 9.4 or R 4.1.2. 

## Results

The study sample size included 60 charts for patients receiving care at the Pulaski Health and Rehabilitation Center between October 17, 2020, and December 30, 2020. Inclusion criteria for the retrospective chart review included patients diagnosed with and treated for COVID-19. Patients meeting the inclusion criteria were divided into an integrative treatment group consisting of 22 patients who were provided the integrated treatment and 38 control/nonintegrative group who were provided treatment using a nonintegrative approach. Exclusion criteria included no COVID-19 diagnosis. Patient charts meeting inclusion criteria were assigned a number; the only recorded information was patient age, mortality, and physician notes indicating symptomatic status or coinfection; no other identifying data were recorded. The charts were retrospectively reviewed, and calculations for mortality were performed. Data analysis revealed a significant reduction in mortality of the integrative group with a p-value of <0.0001. Demographic data are seen in Table 1. For full group data, see Tables 2-5.

**Table 1:** Demographics, risk scores by treatment Epigallocatechin-3-gallate (EGCG): treatment group consisting of EGCG, humic/ fulvic acid, and vitamin C; No EGCG: control group. There was no statistical difference in mortality and risk calculations between groups. The p-value of 0.0589 indicates a more elderly population in the integrative group. Note that the original calculations for mortality and risk are no longer available. References for associated literature and calculations are added: [10-12]

	Group n = 60		p-value
	EGCG (n = 22)	No EGCG (n = 38)	
	Mean ± standard deviation	Mean ± standard deviation	
Age	83.36 ± 8.73	78.07 ± 11.21	0.0589
Mortality risk score	0.41 ± 0.14	0.34 ± 0.21	0.1057
Risk	8.01 ± 4.98	7.01 ± 7.2	0.1158

**Table 2:** Mortality for integrative and nonintegrative groups EGCG: the treatment group consists of epigallocatechin-3-gallate (EGCG), humic/ fulvic acid, and vitamin C; no EGCG:  control group A p-value of <0.0001 reveals a significant reduction in mortality in the integrative group

	Group n = 60		p-value
	EGCG (n = 22)	No EGCG (n = 38)	
Mortality	0%	55%	<0.0001

**Table 3:** Deceased group (N = 21) The age range was 55-100; the mean age was 79 years; the mortality risk mean was 34; and the risk was 6.85%

Patient Index	Age	Calculated Risk of mortality (%)	Risk calculation	Notes/comments
1	75	20%	4	
2	91	52%	7.1	
3	95	40%	5.6	
4	70	34%	3.1	
5	77	75%	14.5	
6	87	81%	5.8	
7	75	31%	3.9	
8	82	52%	7	
9	74	40%	1.3	
10	77	51%	3.0	
11	75	28%	1.9	
12	90	50%	12.8	
13	86	44%	14.4	
14	88	31%	25.9	
15	75	18%	15.2	
16	82	54%	2.8	
17	91	47%	26.5	
18	66	12%	2.5	
19	100	36%	7.1	
20	55	2%	0.6	* Note COVID and influenza
21	66	12%	2.5	

**Table 4:** EGCG group (N = 22 subjects with no deaths) Epigallocatechin-3-gallate (EGCG): treatment group consisting of EGCG, humic/ fulvic acid, and vitamin C The age range was 68-95 years, and the mean was 83 years. The calculated mortality was 38% or eight deaths with a risk of 8.2%. Asymptomatic patients were those identified by weekly screening, with the date of the positive test, who did not develop symptoms. The p-value of 0.0589 indicates a more elderly population in the integrative group. Note that the original calculations for mortality and risk are no longer available References: [10-12]

Patient Index	Age	Calculated risk of mortality (%)	Risk calculation	Notes/comments
22	92	56%	7	11/16 asymptomatic
23	88	31%	6.1	11/24 asymptomatic
24	80	50%	10.7	11/22 asymptomatic
25	66	25%	13.3	11/19 fever 11/24 antibiotics 3d
26	83	50%	8	11/22 asymptomatic
27	78	44%	7	11/21 asymptomatic
28	93	67%	10	11/5 asymptomatic
29	94	50%	10	10/28 asymptomatic
30	68	11%	1.4	11/1 asymptomatic
31	82	31%	2.2	11/13 asymptomatic
32	78	36%	2.3	11/13 asymptomatic
33	85	47%	8.5	11/22 asymptomatic
34	87	51%	19.3	11/19 10 days pneumonia with antibiotics
35	74	21%	10.1	12/3 asymptomatic
36	91	47%	9.6	11/17 asymptomatic
37	82	33%	2.9	12/3 asymptomatic
38	68	40%	7.1	11/2 asymptomatic
39	95	52%	19.7	11/13 asymptomatic
40	93	40%	9.2	11/22 asymptomatic
41	82	43%	2.9	12/3 asymptomatic
42	84	25%	2	10/28 asymptomatic
43	91	62%	7	11/22 pneumonia with 8 days of antibiotics

**Table 5:** Resolved non-EGCG (n = 17) EGCG: Epigallocatechin-3-gallate The age range was 46-90 years with a mean of 68 years; calculated mortality was 28% with 4.6% risk

Patient Index	Age	Calculated risk of mortality (%)	Risk calculation	Notes/comments
44	81	31%	2.0	11/17 asymptomatic
45	74	15%	1.2	11/4 asymptomatic
46	83	41%	4.0	11/22 asymptomatic
47	90	13%	0.6	11/1 asymptomatic
48	80	37%	15.3	11/19 asymptomatic
49	58	5%	0.98	11/4 asymptomatic
50	79	23%	2.9	11/14 asymptomatic
51	70	10%	1.9	11/14 asymptomatic
52	79	49%	8.06	11/1 asymptomatic
53	80	62%	13.2	11/1 asymptomatic
54	64	9%	1.9	11/19 asymptomatic
55	88	31%	25.9	11/11 lost to follow-up admitted afib
56	79	15%	1.4	11/23 sporadic intake EGCG asymptomatic
57	46	1%	0.32	11/1 asymptomatic
58	69	30%	1.3	10/27 asymptomatic
59	82	82%	4.1	11/30 > 10 days pneumonia
60	88	40%	8.4	11/6 > 10 days pneumonia

## Discussion

Mechanisms

**Figure 1:**
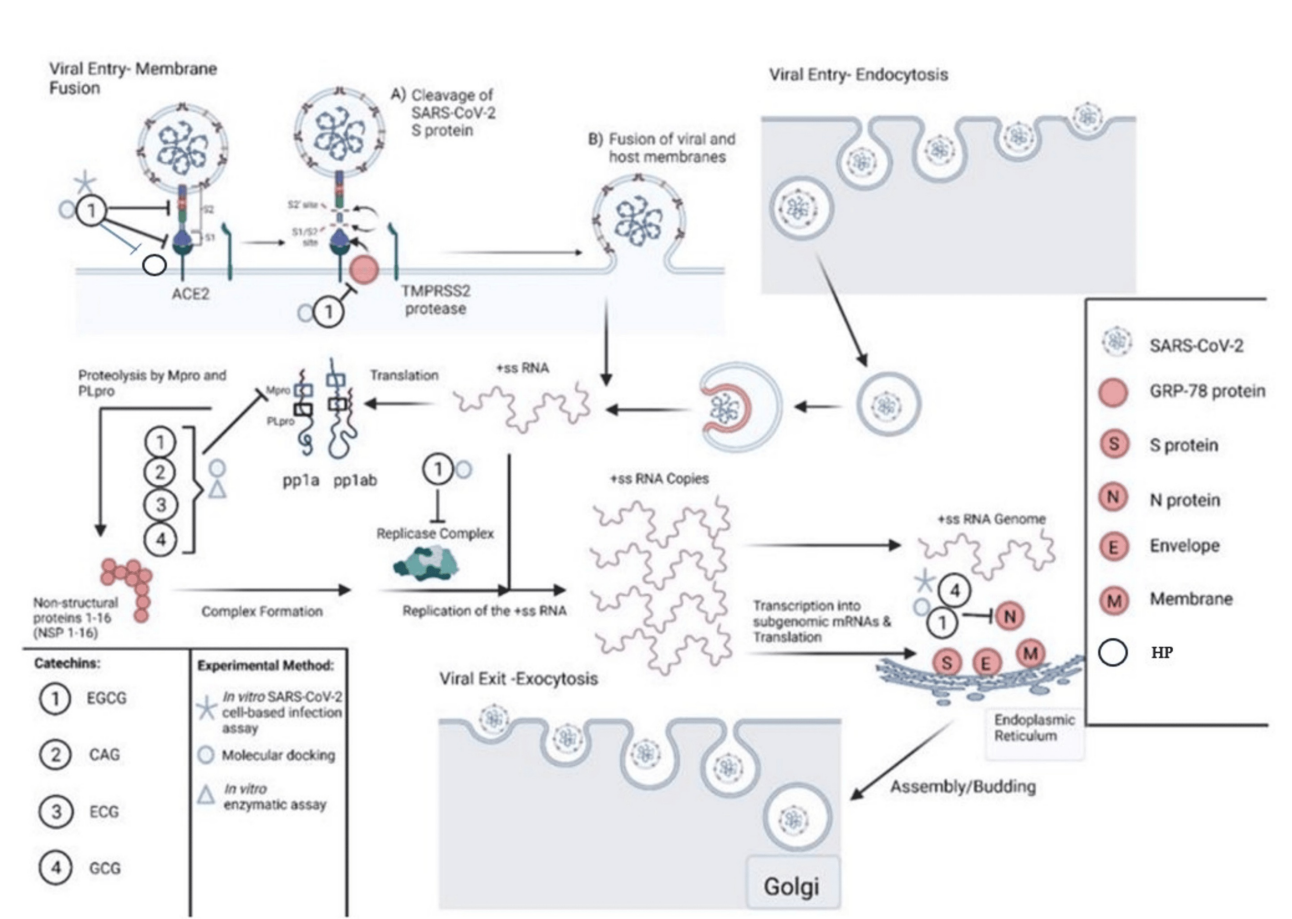
COVID-19 Pathophysiology and EGCG Interaction Catechins, such as epigallocatechin-3-gallate (EGCG), catechin gallate (CAG), epicatechin-3-gallate (ECG), and gallocatechin gallate (GCG), bind to SARS-CoV-2 S protein, inhibiting its binding to the angiotensin-converting enzyme 2 (ACE2) receptor by utilizing transmembrane serine protease 2 (TMPRSS2). EGCG also binds to glucose-regulated protein 78 (GRP78), potentially blocking its binding to spike protein (S protein), along with blocking heparin sulfate proteoglycans (HP), which are needed as a cofactor with ACE2 for host membrane fusion [14]. Catechins inhibit the main protease (Mpro) of SARS-CoV-2, which blocks viral translation via protein phosphatase (pp1a and ab) and subsequent replication. Molecular docking studies have shown that EGCG binds to RNA-dependent RNA polymerase and other proteins of the replicase complex nonstructural proteins (NSSP3, NSP6, and NSP15), which may block viral replication. Furthermore, EGCG and GCG bind to and inhibit the association of nucleocapsid protein (N-protein) with the RNA genome, blocking viral assembly. Adapted from Diniz [15]. Access by Creative Commons

COVID-19 infection follows a defined sequence of events from viral cellular uptake. Cellular uptake is followed by viral reproduction, subsequent inflammatory and immunological responses, and occasional thrombotic/fibrotic events [13]. Humic/fulvic acid and EGCG are naturally occurring agents that can inhibit the above pathophysiologic responses in many viruses, specifically COVID-19; they have excellent safety profiles and are readily available (Figure 1).

Cellular Uptake and Reproduction

**Figure 2:**
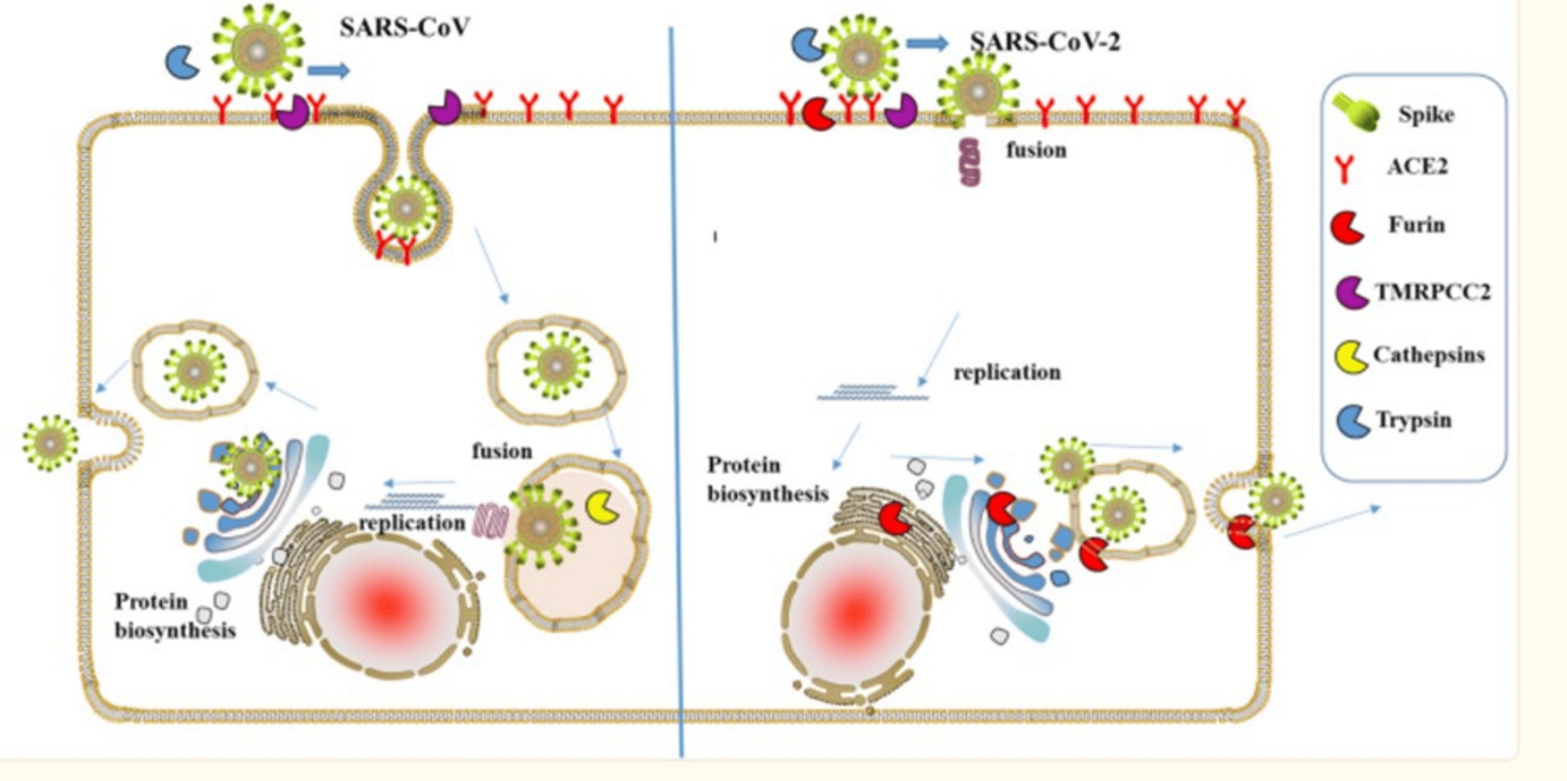
Furin involvement in SARS-CoV attachment and replication "A schematic diagram of the process of severe acute respiratory syndrome coronavirus (SARS-CoV and SARS-CoV-2)-infecting host cells. Those proteases are presented by sector in different colors. The uptake of COVID-19 into the cell involves the interaction of the angiotensin-converting enzyme 2 (ACE2) receptor and COVID-19 spike protein. Furin can cleave spike in the process of viral maturation and enhance receptor affinity and membrane fusion. Epigallocatechin-3-gallate (EGCG) inhibits ACE2, furin, transmembrane protease (TMRPC)C2, and cathepsin interaction/binding [16]." Adapted from [17]. Copyright: iScience open access

Humic acid exhibits viral inhibitory activity in RNA and DNA viruses, including respiratory viruses such as respiratory syncytial virus (RSV) and influenza. Humic/fulvic has been shown to inhibit severe acute respiratory syndrome coronavirus 2 (SARS-CoV-2 and the variants of concern (VOC) Alpha, Beta, Gamma, Delta, and Omicron reproduction even at picomolar concretions [3]. Green tea beverage (GTB) or its major constituent, EGCG, is highly effective in inhibiting infection of live SARS-CoV-2 and human coronavirus (HCoV OC43) in cell culture models. In addition, cell infection by new variants (UK-B.1.1.7, SA-B.1.351, and CA-B.1.429) was prevented by binding of EGCG to viral spike protein 1 adapter protein and ACE cellular receptor sites [16]. The increased infectivity of COVID-19 is proposed to be due to the furin activity of the S1 protein, which EGCG effectively blocks (Figure 2) [17].

EGCG also diminished the binding affinity of the full-length SARS-CoV-2 spike protein to ACE2 [16]. Heparin sulfate proteoglycans are a required attachment factor for SARS-CoV-2 and are known to be important in HCoV-OC43 infection. EGCG can compete with heparin, a heparin sulfate analog, for virion binding. Results highlight heparin sulfate as a conserved cell attachment factor for coronaviruses and demonstrate the potential for developing pan-coronavirus attachment inhibitors [14]. As such, EGCG could be useful to protect against future emerging coronaviruses. 3CL-protease activity of HCoV-OC43 and HCoV-229E decreased in a dose-dependent manner after EGCG treatment, and EGCG also decreased coronavirus-induced cytotoxicity [18]. These studies confirm that EGCG inhibits coronavirus uptake and replication. Another factor in COVID-19 uptake in both cells and the endoplasmic reticulum is glucose-regulated protein 78 (GRP78). This protein is notably elevated in COVID-19 patients with advanced disease, and the COVID-19 virus can also be utilized as an alternative cellular uptake via clathrin adapter protein [19]. EGCG exhibited GRP78-mediated uptake inhibition [20,21].

Immune Response Enhancement and Balancing

EGCG has also been shown to exhibit activity as a vaccine adjuvant and may help improve the response to the COVID-19 vaccine [22]. Nuclear factor erythroid 2-related factor (Nrf2) activators can maintain cells’ redox balance and reduce inflammation. Nrf2 groups of plant-derived chemicals and their analogs might provide a class of drugs with both antiviral and anti-inflammatory properties. It might help tackle the pathophysiological changes in viral pneumonia and acute respiratory distress syndrome (ARDS) [23]. EGCG can restore natural immunological homeostasis in many different autoimmune diseases. Therefore, supplementation therapy with EGCG in COVID-19 patients could be beneficial [24]. Chronic COVID-19 syndrome has also been linked to Nrf2 inhibition and proposed as a treatment avenue for both chronic and acute infections [25].

Anti-inflammatory Properties

Patients with COVID-19 disease have increased proinflammatory cytokines; these include interleukin (IL)-6, IL-10, granulocyte-colony stimulating factor (G-CSF), monocyte chemoattractant protein 1 (MCP1), macrophage inflammatory protein (MIP)1α, and tumor necrosis factor (TNF)-α [26]. “Prostaglandin and leukotriene production contribute to the post-inflammatory response to COVID-19 infections” [27]. Complement activation is also a significant cause of COVID-19 infection [28]. Humic/fulvic complex has complement fixation properties that should be helpful with inflammatory responses. Humic acid significantly inhibited the release of TNF-α, IL-1β, IL-6, and IL-10 by conditioned medium (CM) from phytohemagglutinin (PHA) stimulated mononuclear leucocytes (MNLs). PHA-stimulated MNL inhibits the synthesis of inflammation mediators-prostaglandins. Hyaluronidase accelerates wound healing and is activated locally [29]. Humic acids were shown to inhibit proteolytic enzymes that damage the walls of the vessels and the skin (Table 6) [5].

**Table 6:** Inhibition of inflammatory response to COVID-19 by humic/fulvic acid and EGCG EGCG: epigallocatechin-3-gallate Humic/fulvic complex has complement fixation properties that should be helpful with inflammatory responses. Humic acid significantly inhibits the release of tumor necrosis factor-alpha (TNF-α), interleukins (IL), IL-1β, IL-6, and IL-10 from stimulated mononuclear leucocytes (MNLs) and also inhibits proteolytic enzymes in vessel inflammation. EGCG  can attenuate the inflammatory response in sepsis and respiratory infections and is associated with the release of high mobility group box-1 protein (HMGB1). EGCG can also reduce the release of HMGB1 induced by endotoxins. Serum interleukin (IL-6) remains the best available biomarker to monitor the severity of COVID-19; EGCG significantly reduces this marker in COVID-19 infections. TNF-α, inducible nitric oxide synthase (iNOS), cyclooxygenase-2 (COX-2), and poly(ADP-ribose) polymerase (PARP) are reduced by EGCG/green tea extract (GTE). Their supplementation should be advantageous because of their multitarget action as regulators such as TNF-α, interferon (IFN)-γF-κB. Nuclear factor kappa-light-chain-enhancer of activated B cells (Nrf2) is also decreased by EGCG

EGCG/ humic acid	Inhibition	Reference
Humic/fluvic	Completement fixation TNF-α, IL-1β, IL-6, IL10, prostaglandins	[5,28}
Humid/fulvic	Inhibits proteolytic enzymes in the walls of vessels	[3]
EGCG	Reduces cytoplasmic HMGB1 levels in endotoxin-stimulated macrophages	[29]
EGCG	Myeloperoxidase activity significantly decreased; attenuated TNF-α, IL-1β, nitrotyrosine, iNOS, COX-2, and PARP expression with attenuated myelin degradation. IL-6, INF-kB, Nrf2	[14,28]

**Figure 3:**
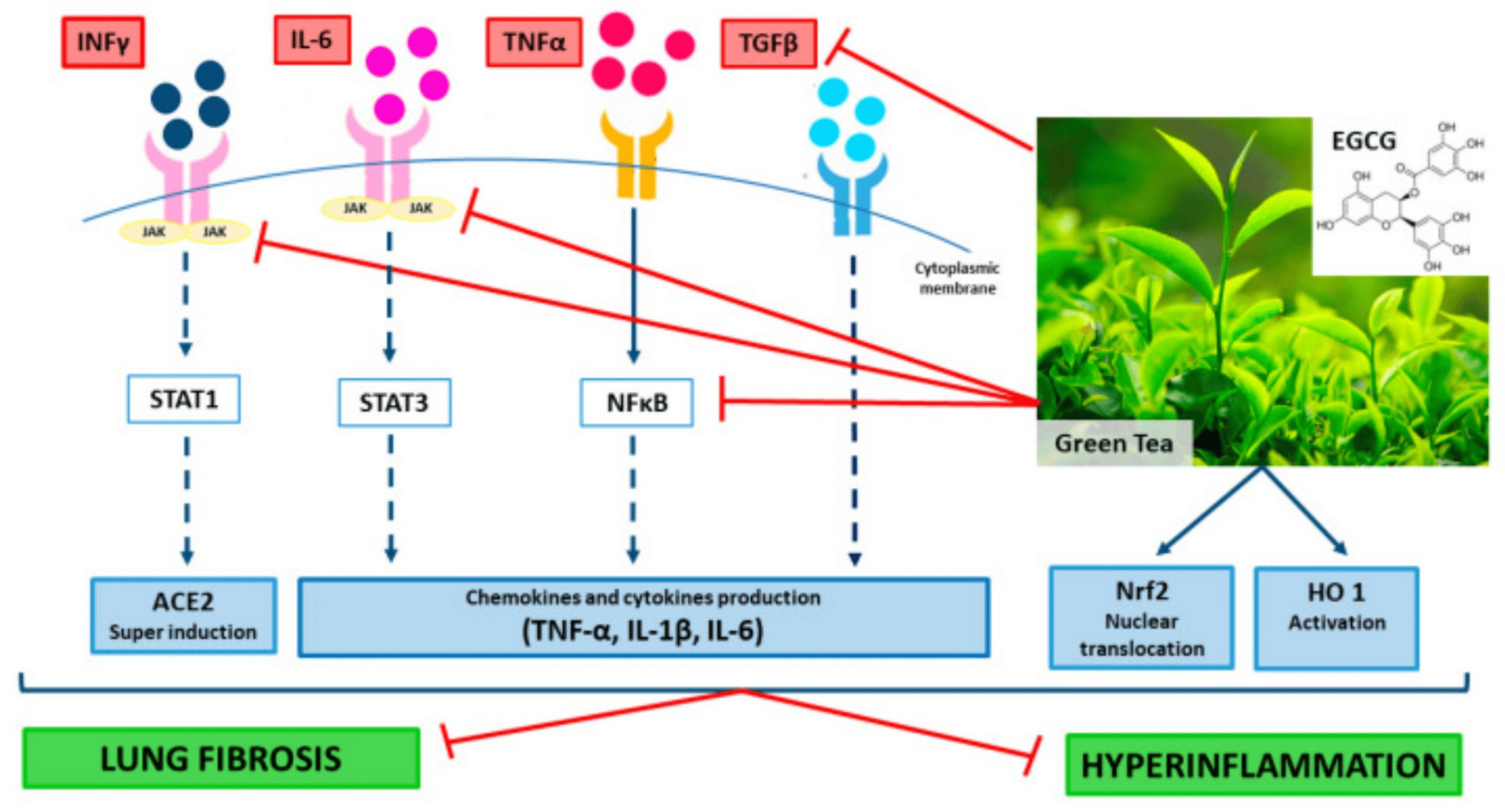
Mechanisms of the potential beneficial effects of green tea extract and epigallocatechin-3-gallate (EGCG) in patients with coronavirus disease (COVID-19) EGCG/green tea extract (GTE) supplementation should be advantageous because of their multitarget action as regulators of both signal transducer and activator of transcription (i.e., STAT1, STAT3), tumor necrosis factor (TNF)-α, interferon (IFN)-γF-κB, nuclear factor kappa-light-chain-enhancer of activated B cells (Nrf2), interleukin(IL) IL-1β, IL-6, heme oxygenase (HO) activities, and expression of their target genes. Adapted from [23]. This article is an open-access article distributed under the terms and conditions of the Creative Commons Attribution (CC BY) license (http://creativecommons.org/licenses/by/4.0/)

EGCG can attenuate the progression of sepsis; the rapid progression in sepsis is associated with the release of high mobility group box-1 protein (HMGB1). EGCG stimulates autophagy and reduces cytoplasmic levels in endotoxin-stimulated macrophages. GTE containing EGCG can also reduce the release of HMGB1 induced by endotoxins. HMGB1 was completely inhibited in macrophages with no evidence of cytotoxicity at 10 μg/mL GTE. EGCG is a potential safe and natural supplement to counteract hyper-inflammation in COVID-19. IL-6 remains the best available biomarker to monitor the severity of COVID-19; it has great potential for guiding disease treatment. EGCG significantly reduces this marker in COVID-19 infections: TNF-α, iNOS, COX-2, and PARP (Figure 3) [27].

SARS-CoV-2 Membrane Disruption

The SARS-CoV-2 viral membrane formation has been proposed as an important factor in infectivity and a target of treatment [17]. EGCG blocks this membrane formation in SARS-CoV-2 via Furin inhibition. See Figure 2.

Anticoagulant Effect

The anticoagulant effect of humic/fulvic acid prevented death caused by pulmonary thrombosis in mice in vivo in a dose-dependent manner, and they inhibited human platelet aggregation in vitro dose-dependently [30]. EGCG has also noted anticoagulant activity [31].

Pulmonary Fibrosis

A proportion of patients who develop ARDS die from progressive pulmonary fibrosis. Significant fibrosis mediators include matrix metalloproteinases, vascular endothelial growth factor (VEGF), and cytokine release that induces epithelial and endothelial injury [16,32]. Data suggest that IL-6 secretion contributes, together with TNF-α, to the development of the disease [23]. After treatment with GTE, the mRNA and protein levels of TNF-α and IL-6 were reduced by 70% and 80%, respectively, thus suggesting that green tea has significant preventive effects on TNF-α-related diseases. EGCG inhibited fibroblast activation and collagen accumulation by downregulating TGF-β1 signaling [33]. “Oral administration of GTE in drinking water to mice significantly reduces pulmonary fibrosis” [34]. 

Limitations

The levels of EGCG in the serum after ingestion are important in the treatment of COVID-19 infection and are influenced by different constituents such as vitamin C. Also, the influence and interaction between EGCG and humic/fulvic acid are unknown. The levels of EGCG were not measured and would help define specifics about COVID-19 and EGCG with humic/fulvic acid uptake versus RNA replication in further studies. There was no defined nonintegrative protocol due to the retrospective study. Future studies should have a specific protocol for both groups, which would reduce possible treatment-induced consequences.

Future considerations

Studies have shown a significant inhibition of surface binding and prevention of virus uptake with both EGCG and humic/fulvic acid. These properties could provide an avenue for COVID-19 prevention in high-risk populations. With the ability to reduce dysregulation secondary to COVID-19 autoimmunization, as noted by sustaining Nrf-2 levels and more balanced TH1/TH2, this combination therapy may also be utilized to reduce associated autoimmune and immunization sequelae such as myocarditis. Now that medications have been approved, this protocol could be utilized in patients unable to take them, secondary to interactions with their current prescribed medical regime.

## Conclusions

The initial mortality of the COVID-19 epidemic approached 50% in the nursing home environment. There were no approved therapeutic regimens in the initial phase of the pandemic, which resulted in individual regimes for treatment. Incorporating a combination of EGCG, vitamin C, and humic/fulvic acid resulted in a significant reduction in COVID-19 mortality in high-risk populations. This paper also provides evidence-based practice (EBP) results for the utilization and further studies of EGCG and humic/fulvic acid in COVID-19 infections.

## References

[1] (2023). The COVID tracking project. https://covidtracking.com/..

[2] Mhatre S, Naik S, Patravale V (2021). A molecular docking study of EGCG and theaflavin digallate with the druggable targets of SARS-CoV-2. Comput Biol Med.

[3] Hajdrik P, Pályi B, Kis Z (2022). In vitro determination of inhibitory effects of humic substances complexing Zn and Se on SARS-CoV-2 virus replication. Foods.

[4] Hafez M, Popov AI, Zelenkov VN (2020). Humic substances as an environmental- friendly organic wastes potentially help as natural anti-virus to inhibit COVID-19. SA.

[5] Jooné GK, van Rensburg CE (2004). An in vitro investigation of the anti-inflammatory properties of potassium humate. Inflammation.

[6] Nagle DG, Ferreira D, Zhou YD (2006). Epigallocatechin-3-gallate (EGCG): chemical and biomedical perspectives. Phytochemistry.

[7] (2024). Vitamin C. https://ods.od.nih.gov/factsheets/VitaminC-HealthProfessional/..

[8] Chen L, Wang W, Zhang J, Cui H, Ni D, Jiang H (2021). Dual effects of ascorbic acid on the stability of EGCG by the oxidation product dehydroascorbic acid promoting the oxidation and inhibiting the hydrolysis pathway. Food Chem.

[9] Bhimraj A, Morgan RL, Shumaker AH (2020). Infectious Diseases Society of America Guidelines on the treatment and management of patients with COVID-19. Clin Infect Dis.

[10] (2024). COVID-19 prognostic tool. https://reference.medscape.com/calculator/731/covid-19-prognostic-tool..

[11] Jehi L, Ji X, Milinovich A (2020). Development and validation of a model for individualized prediction of hospitalization risk in 4,536 patients with COVID-19. PLoS One.

[12] Jin J, Agarwala N, Kundu P, Harvey B, Zhang Y, Wallace E, Chatterjee N (2021). Individual and community-level risk for COVID-19 mortality in the United States. Nat Med.

[13] Liu Q, Gao Y, Ci X (2019). Role of Nrf2 and its activators in respiratory diseases. Oxid Med Cell Longev.

[14] LeBlanc EV, Colpitts CC (2022). The green tea catechin EGCG provides proof-of-concept for a pan-coronavirus attachment inhibitor. Sci Rep.

[15] Diniz LR, Elshabrawy HA, Souza MT, Duarte AB, Datta S, de Sousa DP (2021). Catechins: therapeutic perspectives in COVID-19-associated acute kidney injury. Molecules.

[16] Liu J, Bodnar BH, Meng F (2021). Epigallocatechin gallate from green tea effectively blocks infection of SARS-CoV-2 and new variants by inhibiting spike binding to ACE2 receptor. Cell Biosci.

[17] Wu C, Zheng M, Yang Y (2020). Furin: a potential therapeutic target for COVID-19. iScience.

[18] Jang M, Park R, Park YI, Cha YE, Yamamoto A, Lee JI, Park J (2021). EGCG, a green tea polyphenol, inhibits human coronavirus replication in vitro. Biochem Biophys Res Commun.

[19] Bayati A, Kumar R, Francis V, McPherson PS (2021). SARS-CoV-2 infects cells after viral entry via clathrin-mediated endocytosis. J Biol Chem.

[20] Adachi S, Nagao T, To S (2008). (-)-Epigallocatechin gallate causes internalization of the epidermal growth factor receptor in human colon cancer cells. Carcinogenesis.

[21] Huang HC, Tao MH, Hung TM, Chen JC, Lin ZJ, Huang C (2014). (-)-Epigallocatechin-3-gallate inhibits entry of hepatitis B virus into hepatocytes. Antiviral Res.

[22] Cheong Y, Kim M, Ahn J (2021). Epigallocatechin-3-Gallate as a Novel Vaccine Adjuvant. Front Immunol.

[23] Menegazzi M, Campagnari R, Bertoldi M, Crupi R, Di Paola R, Cuzzocrea S (2020). Protective effect of epigallocatechin-3-gallate (EGCG) in diseases with uncontrolled immune activation: could such a scenario be helpful to counteract COVID-19?. Int J Mol Sci.

[24] Cuadrado A, Pajares M, Benito C (2020). Can activation of NRF2 be a strategy against COVID-19?. Trends Pharmacol Sci.

[25] Dinda B, Dinda S, Dinda M (2023). Therapeutic potential of green tea catechin, (-)-epigallocatechin-3-O-gallate (EGCG) in SARS-CoV-2 infection: major interactions with host/virus proteases. Phytomed Plus.

[26] Van Rensburg CE, Badenhorst BE, Gandy JJ (2010). Potassium humate reduces inflammation and clinically improves the outcomes of patients with osteoarthritis of the knee. Open Conf Proc J.

[27] Lu FJ, Tseng SN, Li ML, Shih SR (2002). In vitro anti-influenza virus activity of synthetic humate analogues derived from protocatechuic acid. Arch Virol.

[28] Wang YQ, Li QS, Zheng XQ, Lu JL, Liang YR (2021). Antiviral effects of green tea EGCG and its potential application against COVID-19. Molecules.

[29] Sriram N, Kalayarasan S, Manikandan R, Arumugam M, Sudhandiran G (2015). Epigallocatechin gallate attenuates fibroblast proliferation and excessive collagen production by effectively intervening TGF-β1 signalling. Clin Exp Pharmacol Physiol.

[30] Lan HT, Zheng YT, Tong ZJ, Zhang C, Cong XY, Wang ZH (2022). Humic acids inhibit platelet activation to reduce venous thromboembolism in mice. Evid Based Complement Alternat Med.

[31] Kang WS, Lim IH, Yuk DY (1999). Antithrombotic activities of green tea catechins and (−)-epigallocatechin gallate. Thrombosis Research.

[32] Schepetkin I, Khlebnikov A, Kwon BS (2002). Medical drugs from humus matter: focus on mumie. Drug Development Research.

[33] Bimonte S, Forte CA, Cuomo M, Esposito G, Cascella M, Cuomo A (2021). An overview on the potential roles of EGCG in the treatment of COVID-19 infection. Drug Des Devel Ther.

[34] Zhang Z, Zhang X, Bi K, He Y, Yan W, Yang CS, Zhang J (2021). Potential protective mechanisms of green tea polyphenol EGCG against COVID-19. Trends Food Sci Technol.

